# Phenotypic, cytogenetic, and molecular marker analysis of *Brassica napus* introgressants derived from an intergeneric hybridization with *Orychophragmus*

**DOI:** 10.1371/journal.pone.0210518

**Published:** 2019-01-10

**Authors:** Chuanyuan Xu, Qian Huang, Xianhong Ge, Zaiyun Li

**Affiliations:** National Key Laboratory of Crop Genetic Improvement, Huazhong Agricultural University, Wuhan, P. R. China; Chungnam National University, REPUBLIC OF KOREA

## Abstract

Aneuploids of a single species that have lost or gained different chromosomes are useful for genomic analysis. The polyploid nature of many crops including oilseed rape (*Brassica napus*) allows these plants to tolerate the loss of individual chromosomes from homologous pairs, thus facilitating the development of aneuploid lines. Here, we selected 39 lines from advanced generations of an intergeneric hybridization between *Brassica rapa* and *Orychophragmus violaceus* with accidental pollination by *B*. *napus*. The lines showed a wide spectrum of phenotypic variations, with some traits specific to *O*. *violaceus*. Most lines had the same chromosome number (2n = 38) as *B*. *napus*. However, we also identified *B*. *napus* nulli-tetrasomics with 22 A-genome and 16 C-genome chromosomes and lines with the typical *B*. *napus* complement of 20 A-genome and 18 C-genome chromosomes, as revealed by FISH analysis using a C-genome specific probe. Other lines had 2n = 37 or 39 chromosomes, with variable numbers of A- or C-genome chromosomes. The formation of quadrivalents by four A-genome chromosomes with similar shapes suggests that they were derived from the same chromosome. The frequent homoeologous pairing between chromosomes of the A and C genomes points to their non-diploidized meiotic behavior. Sequence-related amplified polymorphism (SRAP) analysis revealed substantial genomic changes of the lines compared to *B*. *rapa* associated with *O*. *violaceus* specific DNA bands, but only a few genes were identified in these bands by DNA sequencing. These novel *B*. *napus* aneuploids and introgressants represent unique tools for studies of *Brassica* genetics and for *Brassica* breeding projects.

## Introduction

Maintaining a balanced euploid genome is a key feature of all multicellular organisms. However, aberrant segregation events during mitosis or meiosis can result in aneuploidy, a condition in which cells acquire a karyotype that is not a whole-number multiple of the haploid complement. The balance between chromosome types and the genes they encode is disturbed, resulting in the altered expression of many genes [1–5].

The most common viable form of aneuploidy in humans is Down syndrome, which results from the presence of a third copy of chromosome 21 in a diploid background. This syndrome is typically associated with a delay in cognitive ability and physical growth, as well as a particular set of facial characteristics [6]. Plants are more tolerant of aneuploidy than are animals, and aneuploid individuals are frequently found spontaneously within polyploid plant populations. Various types of dosage compensation can affect plant autosomes, allowing them to better tolerate gene copy imbalances [7]. These aneuploids exhibit few or subtle phenotypic abnormalities and can often compete with their euploid progenitors [8]. Plants therefore provide an excellent opportunity for genome-wide investigations of aneuploid syndromes [9–12].

Many plants, including crops, are allopolyploids, that contain two or more genomes from different progenitors. The polyploid nature of these plants, including cereals (e.g., *Triticum aestivum* L., 2n = 6X = 42, AABBDD genomes) and oilseed rape (*B*. *napus* L., 2n = 4X = 38, AACC), allows them to tolerate the loss of individual chromosomes from homologous pairs, thus facilitating the development of aneuploid lines. As described in detail previously [13], the availability of intraspecific aneuploids (including hypoploids such as nullisomics and monosomics, as well as hyperploids such as trisomics and tetrasomics) and interspecific aneuploids (harboring alien chromosome additions or substitutions) has greatly contributed to studies of chromosome homoeology, genomic analysis, and the chromosomal localization of genes [14–20]. However, it is challenging to produce hypoploid s in *Brassica* and few have been reported [4, 5, 21–23]. Nulli-tetrasomics are individuals in which one chromosome pair is missing but four copies of another nonhomologous chromosome are present. In common wheat (*Triticum aestivum*), a complete set of compensating nulli-tetrasomics has been obtained and studied extensively to investigate the homoeologous relationships of chromosomes [14], as the lack of one chromosome pair (nullisomics) can be genetically compensated for by the presence of four copies of a different pair of chromosomes (nulli-tetrasomics). The presence of two extra chromosomes could result in decreased, unchanged, or increased amounts of proteins [24–26].

However, various *Brassica* aneuploids, including hypoploids and those harboring alien additions or substitutions, have been identified among the hybrid progeny from intergeneric crosses between cultivated *Brassica* species in the U-triangle [27] and another crucifer, *Orychophragmus violaceus* (L.) O. E. Schulz (2n = 2X = 24, OO). These lines have been examined for the possible occurrence of complete and partial separation of parental genomes during mitotic and meiotic divisions in the hybrids [13,28–32]. Many novel lines have been established from a single mixoploid hybrid progeny between *Brassica rapa* L. (2n = 2X = 20) and *O*. *violaceus* through successive selections for fertility and viability for 10 generations. The lines with high productivity showed a wide spectrum of phenotypes and seed quality profiles, as well as variations in genomic and chromosomal constituents. These lines had variable chromosome numbers centered on the same number as *B*. *napus* (2*n* = 38) but contained no intact *O*. *violaceus* chromosomes, as revealed by genomic *in situ* hybridization (GISH) analysis [33].

**Fig 1:**
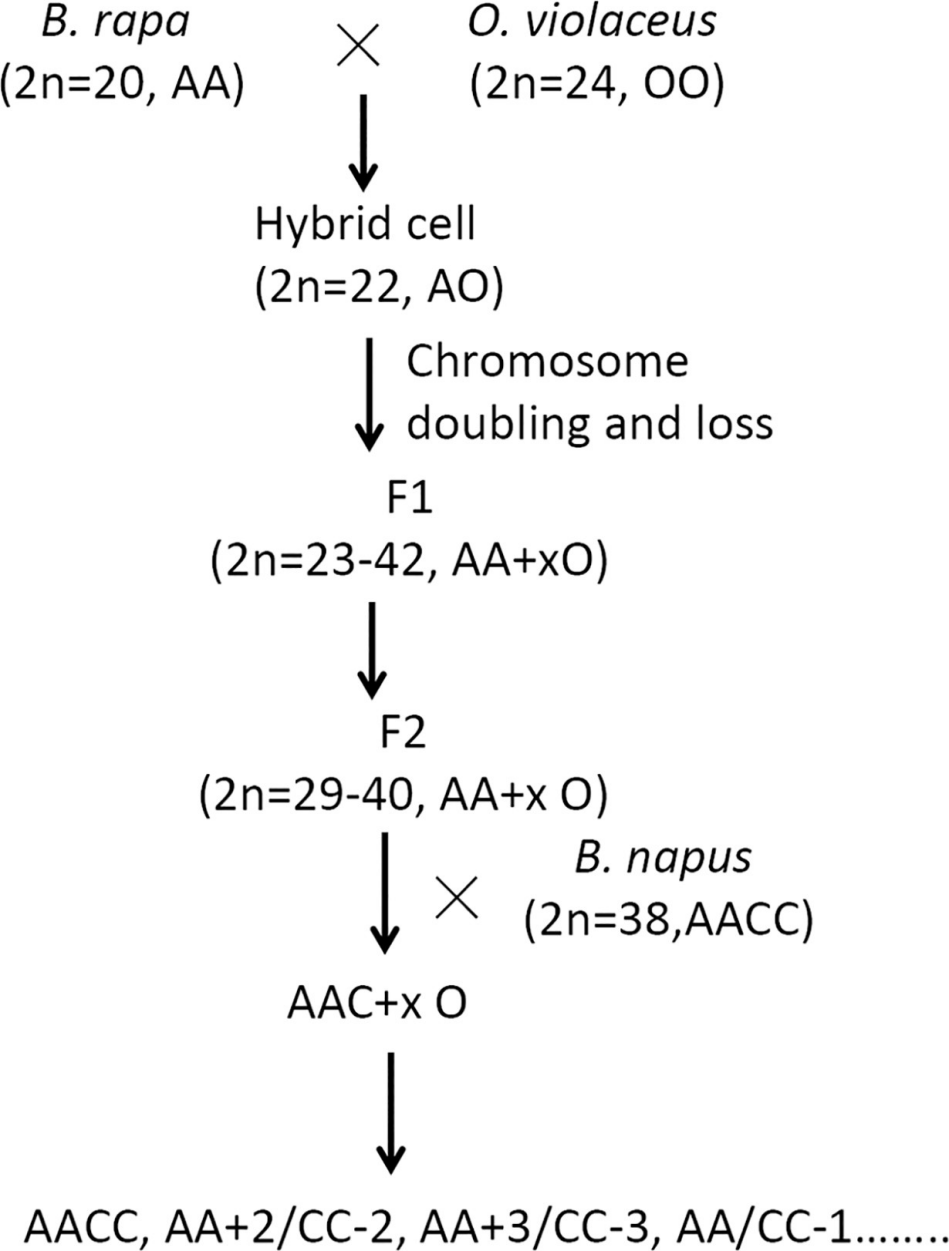
Crossing and selection scheme for new *B*. *napus* types produced from a single hybrid between *B*. *rapa* and *O*. *violaceus*. xO: uncertain number of chromosomes from *O*. *violaceus*.

In this study, through consecutive selections for several generations based on phenotype and fertility, we established 39 F_16_ lines and characterized their phenotypes, genomic/chromosomal complements, and meiotic pairing. Interestingly, several types of chromosomal complements were detected, including *B*. *napus* nulli-tetrasomics with 22 A-genome and 16 C-genome chromosomes, aneuploids with variable A- or C-genome chromosomes, and *B*. *napus* introgressants with 20 A-genome and 18 C-genome chromosomes. These lines showed genomic variations and deviations from parental line *B*. *rapa*, as they exhibited the loss of DNA bands from the *B*. *rapa*, the gain of novel bands, and the introgression of bands from *O*. *violaceus*. These *B*. *napus*-like lines likely originated from the pollination of hybrid progeny by *B*. *napus* and subsequent chromosomal reorganization during the long process of artificial selection (Fig 1). These *B*. *napus* aneuploids and introgressants could serve as valuable tools for *Brassica* breeding efforts and genetic analysis.

## Materials and methods

### Plant materials

An intergeneric cross between *B*. *rapa* cv. ‘Aijiaophuang’ and *Orychophragmus violaceus*, with the latter used as the male parent, was performed by hand emasculations and pollinations in 1995, and only one mixoploid hybrid (2n = 23–42) progeny with some *O*. *violaceus* characteristics was identified [30]. This hybrid was partially fertile and produced some seeds of different sizes after selfing and open pollination. From the 10 F_2_ plants, 59 F_10_ lines were developed by consecutive selection for viability and seed fertility. These lines showed a wide spectrum of phenotypes and some variations in chromosome numbers, but most had 2n = 38 chromosomes [33]. The pedigrees of these lines were advanced to the F_16_ generation by selfing, with further selection for phenotype and seed set. The F_16_ seeds, together with *B*. *rapa* ‘Aijiaohuang’ and *O*. *violaceus* seeds, were planted in an experimental field at Huazhong Agricultural University in October 2010, and the young leaves of F_16_ plants and their two parents were collected for DNA extraction. Young ovaries and floral buds were also collected from the same plant for cytological analysis in the Spring of 2011. Petiole length and petiole angle of three active basal leaves were surveyed at the flowering stage from three plants per line. Some lines were crossed with both *B*. *rapa* ‘Aijiaohuang’ and *B*. *napus* ‘Oro’. The hybrid progeny were planted in October 2011, and floral buds were collected for chromosome pairing observation in the Spring of 2012.

### Cytological investigation and pollen fertility analysis

Ovaries from young flower buds were collected and treated with 8-hydroxyquinoline for 3–4 h at room temperature before being fixed in Carnoy’s solution I (3:1 ethanol:glacial acetic acid, v/v) and stored at –20°C for chromosome counting in somatic cells. The young flower buds were fixed directly in a Carnoy’s solution and stored at –20°C for meiosis studies. Cytogenetic observation was carried out according to the methods as described previously [28]. More than 300 pollen grains from three flowers of the same plant were stained with acetocarmine (1%, w/v), and the percentage of stainable pollen grains was calculated to measure pollen viability.

### DNA extraction, labeling, and *in situ* hybridization

Plasmid DNA of BAC BoB014O06 (*Brassica* C-genome-specific repetitive sequence BAC clone, provided by Dr. Susan J. Armstrong, University of Birmingham, Birmingham, UK) was extracted, labeled with biotin-11-dUTP by random priming using a Bio-Prime DNA Labeling System Kit (Invitrogen, Life Technologies), and used as a probe. Fluorescent *in situ* hybridization was performed following standard procedures [34] with minor modifications. The probe mixture consisted of 50% (v/v) formamide, 2× SSC, 10% (w/v) dextran sulfate, 2 μg salmon sperm DNA, 125 μM EDTA, 0.125% (w/v) SDS, and 50 ng of the labeled probes in a total volume of 50 μL. The probe mixture was pre-denatured at 85°C for 10 min and cooled on ice for at least 5 min. The mixture was placed onto a slide and covered with a plastic coverslip. The probe and preparation were then denatured together at 74°C for 7 min before cooling slowly to 37°C for overnight hybridization. The immunodetection of biotinylated and digoxigenated DNA probe was carried out using Cy3-labeled streptavidin (KPL, St. Louis, MO, USA). Finally, the preparations were counterstained with 49-6-diamidino-2-phenylindole (DAPI) solution (Roche, Basel, Switzerland) (1 mg/mL) and mounted in antifade solution (Vector Laboratories, Peterborough, UK). Images were taken under a Zeiss Axioplan fluorescent microscope with a CCD camera and processed in Photoshop using only functions that affected the entire image equally.

### Sequence-related amplified polymorphism (SRAP) analysis, DNA recovery, and sequencing

Total genomic DNA was extracted and purified from young leaves according to classical methods [35]. Randomly selected SRAP primer pairs were used to detect polymorphisms in open reading frames [36]. Each 20-μL PCR mixture consisted of 1.5 U Taq DNA polymerase (Fermentas), 1× PCR buffer, 0.2 mM dNTP, 0.3 μM primer, 3 mM Mg^2+^, and 25–100 ng template DNA. The thermal cycling conditions were 3 min at 94°C for initial denaturing, five cycles of 30 s at 94°C, 30 s at 35°C, and 1 min at 72°C, followed by 35 cycles of 30 s at 94°C, 30 s at 50°C, and 1 min at 72°C. The last cycle was followed by a 7-min extension at 72°C. Amplified products were analyzed on 8% (w/v) polyacrylamide gels and visualized by silver staining. The 21 SRAP primer pairs were used, and 80- to 800-bp bands were scored.Interesting specific bands from *B*. *rapa* and *O*. *violaceus* and new bands in progeny lines were excised from the gel, squashed, and dissolved in distilled water by boiling for 15 min. The fragments were re-amplified as described for the SRAP protocol. The PCR products were cloned into the pMD18-T vector (TaKaRa), and 3–5 individual clones were sequenced.

## Results

### Phenotypic variation

**Fig 2:**
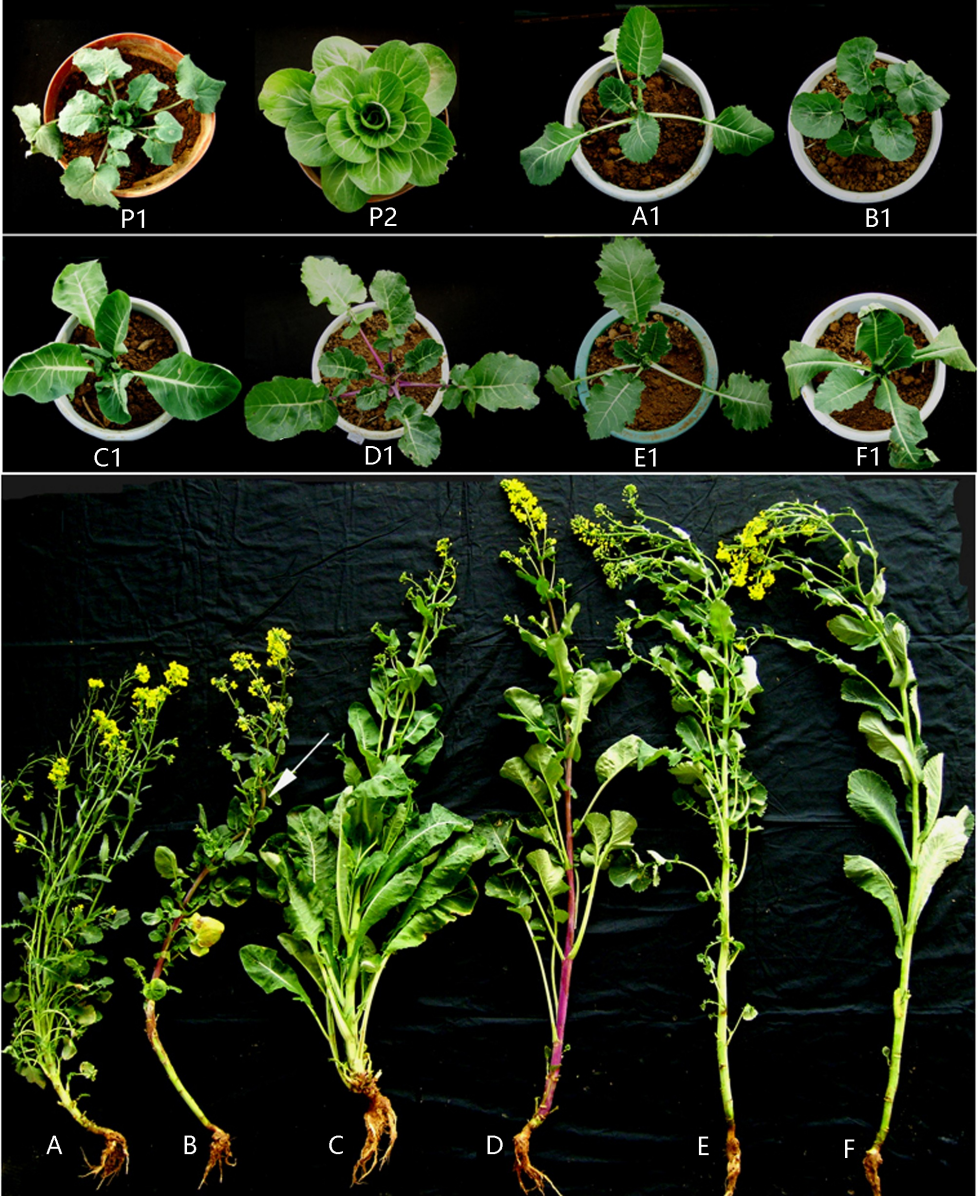
Morphology of the F_16_ lines. Upper panels: young *O*. *violaceus* (P1) and *B*. *rapa* (P2) plants; A1–F1: Lines 20, 17, 10, 2, 25, and 8. Lower panel: (A–F): flowering plants of all six lines. The arrow points to a bent main stem.

**Fig 3:**
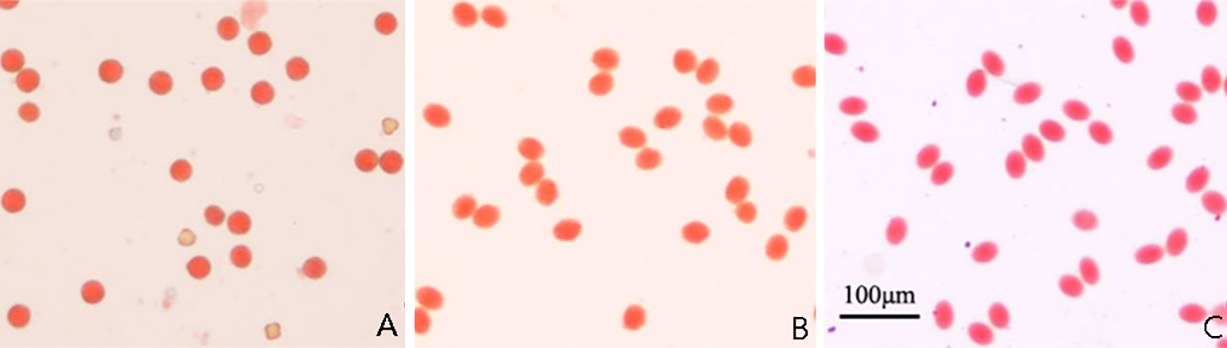
Different pollen grain shapes in the progeny lines. (A) Stainable round and unstainable shrunken pollen grains from Line 5. (B, C) Stainable elliptic pollen grains from Line 23 and *O*. *violaceus*, respectively.

F_10_ lines, derived from a single intergenetic hybridization between *Brassica rapa* cv. ‘Aijiaohuang’ and *Orychophragmus violaceus*, that had distinct phenotypes and good seed set [30, 33] were selected and used to produce 39 F_16_ lines that could be distinguished based on their morphological differences (Fig 1). The spectrum of phenotypes ranged from *B*. *rapa*-type to *O*. *violaceus*-type, with most being intermediate. Several traits that originated from *O*. *violaceus* were readily detected among the lines, such as serrated leaves, purple coloration of stems, leaf veins, and even pods, basic clustering branches, and drooping inflorescences (Fig 2). The differences in leaf phenotype, including leaf shape, lobes, serration, hairs, color, and petiole length and angle (Fig 2; S1 Table), were most obvious. The leaves and stems of Line 2 were deep purple, while Line 21 had drooping stems, like *O*. *violaceus* (Fig 2). Interestingly, some lines produced elliptic pollen grains similar to those of *O*. *violaceus* (Fig 3). These lines also showed wide variations in flowering time after planting (at the beginning of October in Wuhan), ranging from 46 days for Line 25 to 188 days for Line 10 and an average of 129 days. Most lines flowered at similar times to *B*. *rapa* ‘Aijiaohuang’ but earlier than winter type *B*. *napus*, which requires approximately 150 days for flowering. Notably, most lines exhibited phenotypes similar to those of *B*. *napus*, such as waxy powder on the plant surface. Cytological analysis (described below) indicated that these lines contained C-genome chromosomes, which were likely introduced through pollination of the hybrid progeny by *B*. *napus* at a certain stage of selection (Fig 1).

### Chromosomal complements

**Fig 4:**
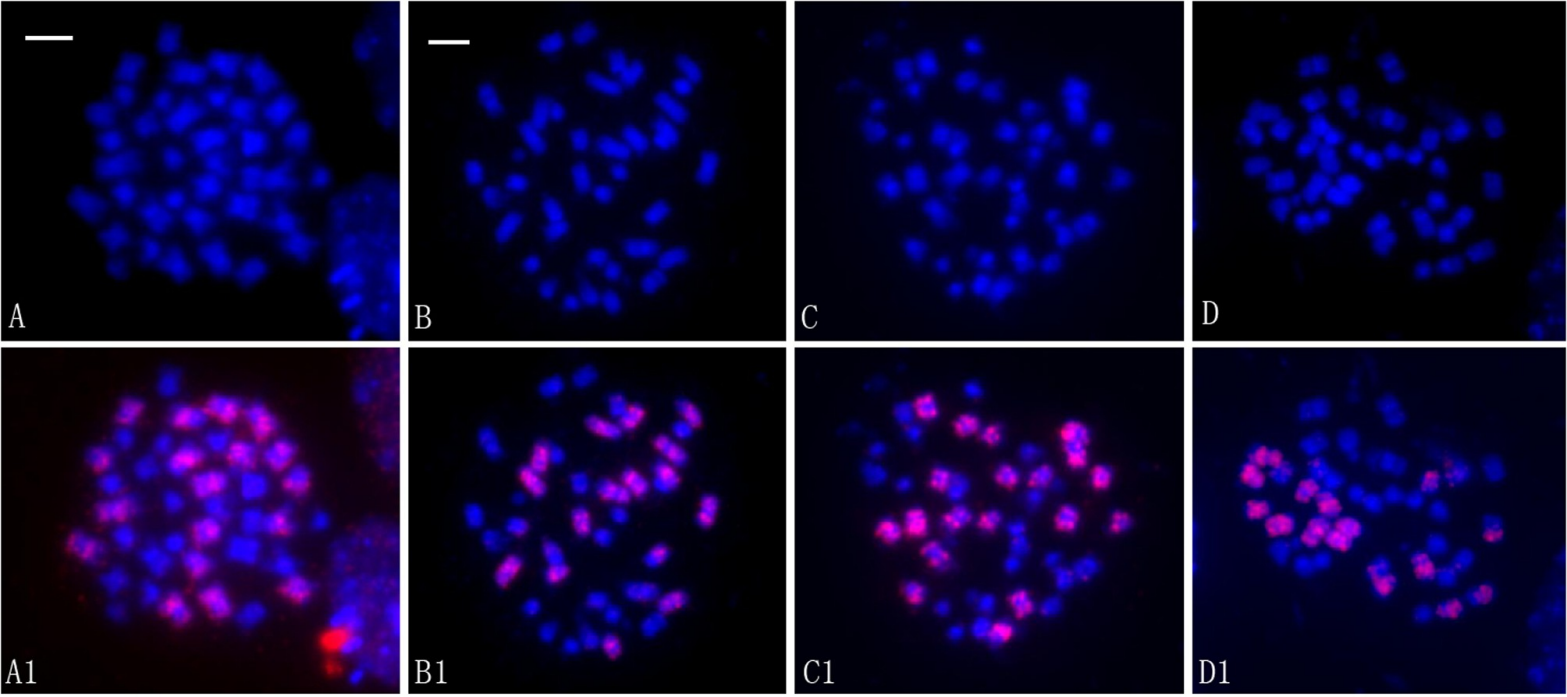
Chromosome complements of different lines, as revealed by BAC-FISH analysis. Red signals are from the C-genome-specific probe; DAPI and merged images are shown for each cell. (A–D) 2n = 38, 38, 39, and 38 from Lines 23, 28, 12, and 4, respectively, which include 18, 16, 19, and 15 labeled chromosomes (A1–D1). Bar: 10 μm.

We determined the somatic chromosome numbers of these lines via conventional cytological analysis and examined the genomic origins of the chromosomes by FISH analysis. Analysis of the chromosome numbers in ovary cells of the 39 F_16_ lines showed that one line (2.6%) had 2n = 37 chromosomes, two (5.1%) had 2n = 39 chromosomes, and 36 (92.3%) had 2n = 38 chromosomes (Table 1). Using labeled genomic DNA from *O*. *violaceus* as a probe, no intact chromosomes were detected in any line, as reported previously [33]. However, using labeled C-genome-specific BAC clones as probes, very clear signals appeared on some chromosomes, indicating their genome origin (Fig 4). Among the 36 lines with 2n = 38 chromosomes, 15 lines contained 20 chromosomes from the A genome and 18 chromosomes from the C genome, i.e., the same complement as that of *B*. *napus*, whereas 19 lines contained 22 chromosomes from the A genome and 16 chromosomes from the C genome (Fig 4A–4A1 and 4B–4B1). Of the two lines with 2n = 39, one had 22 A-genome and 17 C-genome chromosomes and the other had 20 A-genome and 19 C-genome chromosomes (Fig 4C–4C1). The two remaining lines with 2n = 38 contained 23 A-genome and 15 C-genome chromosomes (Fig 4D–4D1). The only plant with 2n = 37 chromosomes contained 20 A-genome and 17 C-genome chromosomes, as it has lost one C-genome chromosome from *B*. *napus*. Therefore, nearly half of the lines (17/39) recovered the chromosome complement of *B*. *napus*, while the remaining half had lost or gained individual chromosomes, mainly gaining A-genome and losing C-genome chromosomes.

**Table 1:** Quadrivalents formation in meiotic cells and pollen viability in various lines.

Lines	2n	Chromosome complement (A/C)	Number of PMC with quadrivalents				Percentage (%) of PMC with quadrivalent	Quadrivalents /PMC	Pollen stainabiliy (%)
			0	1	2	3/4			
**1**	38	22/16	57	17	4	0	26.9	0.3	98.3
**3**	38	22/16	22	15	1	1	43.6	0.5	91.2
**7**	38	22/16	40	12	1	0	24.5	0.3	86.8
**8**	38	22/16	31	9	0	0	22.5	0.2	89.9
**9**	38	22/16	51	28	17	0	46.9	0.6	91.9
**10**	38	22/16	30	9	4	0	30.3	0.4	58.8
**11**	38	22/16	22	17	14	6	62.7	1.1	75.4
**16**	38	22/16	76	31	3	0	30.9	0.3	74.2
**17**	38	22/16	55	12	0	0	17.9	0.2	91.9
**20**	38	22/16	37	14	6	0	35.1	0.5	73.8
**21**	38	22/16	24	39	17	9/2	72.8	1.2	79.6
**22**	38	22/16	87	24	13	2	30.9	0.4	46.3
**24**	38	22/16	35	31	3	0	49.3	0.5	88.5
**28**	38	22/16	51	16	7	0	31.1	0.4	94.2
**32**	38	22/16	55	30	11	3	45.0	0.6	76.6
**33**	38	22/16	81	29	11	0	33.1	0.4	96.8
**35**	38	22/16	70	28	11	5	38.6	0.6	42.2
**37**	38	22/16	40	15	4	0	32.2	0.4	96.7
**38**	38	22/16	35	15	0	0	30.0	0.3	71.6
**Average**			47.3	20.6	6.7	1.4	37.1	0.5	80.2
**6**	38	20/18	36	3	0	0	7.7	0.1	65.2
**13**	38	20/18	36	4	0	0	10.0	0.1	89.6
**14**	38	20/18	80	2	0	0	2.4	0.0	74.9
**15**	38	20/18	50	5	0	0	9.1	0.1	81.8
**18**	38	20/18	86	15	0	0	14.9	0.1	89.7
**19**	38	20/18	/	/	/	/	/	/	94.3
**23**	38	20/18	35	20	3	0	39.7	0.4	94.7
**25**	38	20/18	/	/	/	/	/	/	89.3
**26**	38	20/18	90	6	0	0	6.3	0.1	98.9
**27**	38	20/18	55	3	0	0	5.2	0.1	96.9
**29**	38	20/18	46	4	0	0	8.0	0.1	95.6
**30**	38	20/18	32	1	0	0	3.0	0.0	78.3
**31**	38	20/18	62	2	0	0	3.1	0.0	94.1
**34**	38	20/18	42	2	0	0	4.6	0.0	93.9
**39**	38	20/18	90	7	1	0	8.2	0.1	90.1
**Average**			56.9	5.7	0.31	0	9.4	0.1	88.5
**2**	37	20/17	44	4	0	0	8.3	0.1	88.7
**5**	39	22/17	33	12	4	2	35.3	0.5	90.1
**12**	39	20/19	74	40	5	1	38.3	0.4	75.4
**4**	38	23/15	16	5	0	0	23.8	0.2	84.2
**36**	38	23/15	33	7	0	0	17.5	0.2	90.3
**Average**			40	13.6	1.8	0.6	24.64	0.28	85.74

To examine the genomic relationship of these lines with *B*. *rapa* and *B*. *napus*, we hybridized several selected lines with these two species, which showed high crossability and seed set. The chromosomes in pollen mother cells (PMCs) of the hybrids with *B*. *rapa* were paired as 9–12 bivalents and 5–11 univalents, but those in the hybrids with *B*. *napus* showed relative normal pairing, predominantly as 19 bivalents, or 17 bivalents and one quadrivalent, pointing to the homology of their chromosomes (S1 Fig, S2 Table).

### Chromosome pairing and pollen fertility

We expected that some multivalents, including trivalents and quadrivalents, would form in the PMCs of some lines containing more than two copies of an individual chromosome. We therefore examined chromosome pairing by observing 21–121 PMCs per line at diakinesis, with an average of 65 PMCs, but were unable to analyze Lines 19 and 25 due to the failure to collect enough flower buds at suitable stages. In all lines examined, the chromosomes were mainly paired as bivalents, but there were 0.1–1.1 quadrivalents per PMC, and 2.4–73.3% PMCs contained at least one quadrivalent (Table 1). At the extreme, up to four quadrivalents in one PMC occurred in line 21, with 22 A-genome and 16 C-genome chromosomes. On average, 37.1% of the PMCs had at least one quadrivalent, as detected in 19 lines with 22 A-genome and 16 C-genome chromosomes; this percentage was significantly higher than the 9.4% observed for the 13 lines with 20 A-genome and 18 C-genome chromosomes (χ2 test, P = 2.02E-05<0.01). Trivalents or univalents were often found in five lines with odd numbers of A- or C-genome chromosomes, but rarely in other lines.

**Fig 5:**
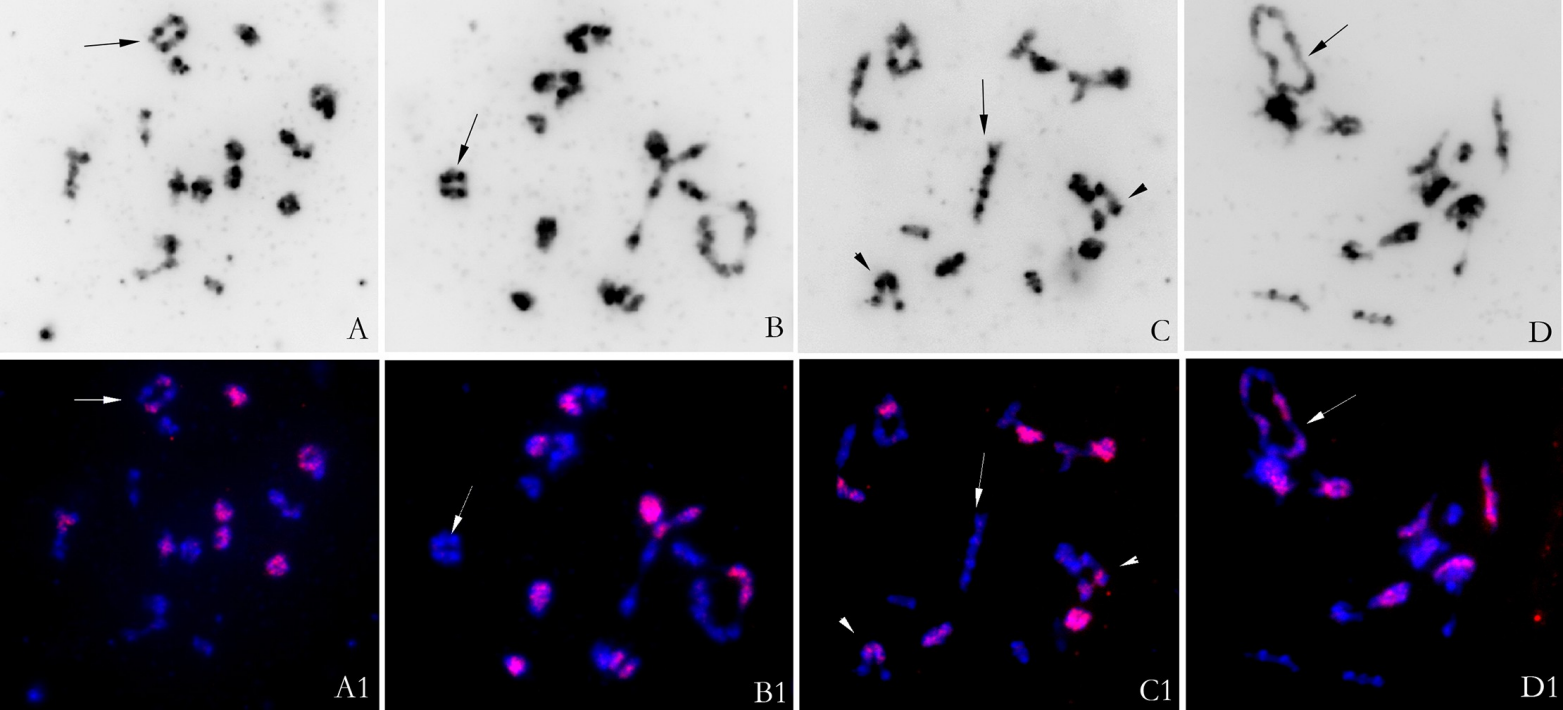
BAC-FISH analysis of meiotic cells from line 21. A–D: Inverted images of DAPI staining. A1–D1: Merged images. Red signals are from the C-genome-specific probe. Arrows show quadrivalents or multivalents. The two arrowheads in C1 show two pairs of homologous chromosomes between A and C genome chromosomes.

In lines with 22 A-genome and 16 C-genome chromosomes, FISH analysis using a C-genome-specific probe revealed the occurrence of quadrivalents formed by one pair of C-genome and A-genome chromosomes (Fig 5A and 5A1) and by four unlabeled A-genome chromosomes (Fig 5B–5B1 and 5C–5C1). The two types of quadrivalents appeared as rings or chains (Fig 5A–5A1, 5C–5C1). The highly similar morphology of the four paired A-genome chromosomes also indicated that they might originat from duplication of the same chromosome (Fig 5B–5B1 and 5C–5C1). Of course, four A chromosomes involved in the tetravalents might also be paralogous A genome chromosomes due to the genome collinearities existing between paralogs. In fact, pairing between paralogous chromosomes are frequent at diakinesis in *Brassica* like in diploids of *B*. *oleracea* [37]. The only way to confirm the origin of the chromosomes would be to use BACs that hybridize specifically to a unique A chromosome pair. Of the 57 cells with clear pairing configurations and bright signals, 43 (75.4%) cells contained at least one quadrivalent, and only 9 (14.5%) of the 62 quadrivalents counted were formed by four A-genome chromosomes, whereas the others were formed by one pair of A-genome and C-genome chromosomes. This finding indicates that the formation of the quadrivalent by four A-genome chromosomes occurs much less frequently than their pairing as two bivalents. Meanwhile, the finding also indicated that homologous pairing of the four A-genome chromosomes occurs less frequently than homoeologous pairing between one pair of A-genome and C-genome chromosomes. Occasionally, we detected multivalents formed by more than four A- and C-genome chromosomes (Fig 5D and 5D1). Bivalents formed by two A- and C-genome chromosomes other than quadrivalents were observed at a very low rate (Fig 5C and 5C1). In the lines with 20 A-genome and 18 C-genome chromosomes, homeologous quadrivalents were only formed by one pair of C-genome and A-genome chromosomes. Among the lines observed by FISH, no quadrivalents formed by four C-genome chromosomes were detected.

The average pollen viability of these lines was 84.1%. Most lines had relatively high fertility, but two lines (Lines 22 and 35) had low fertility, 46.3% and 46.2%, respectively (Table 1). There was a weak, negative correlation between pollen viability and the average number of quadrivalents in PMCs (r = −0.26). However, although the average pollen viability of 19 lines with 22 A-genome and 16 C-genome chromosomes (80.2%) was <87.9% for the 13 lines with 20 A-genome and 18 C-genome chromosomes, this difference was not significant (χ2 test, P = 0.14>0.01), suggesting that their higher frequency of quadrivalent formation did not affect pollen viability. Finally, Lines 22 and 35, with 22 A-genome and 16 C-genome chromosomes, showed much lower fertility than the other lines with such chromosome complements, and they had higher mean numbers of quadrivalents (0.4 for Line 22 and 0.6 for Line 35).

### Genomic composition revealed by SRAP and sequencing

**Fig 6:**
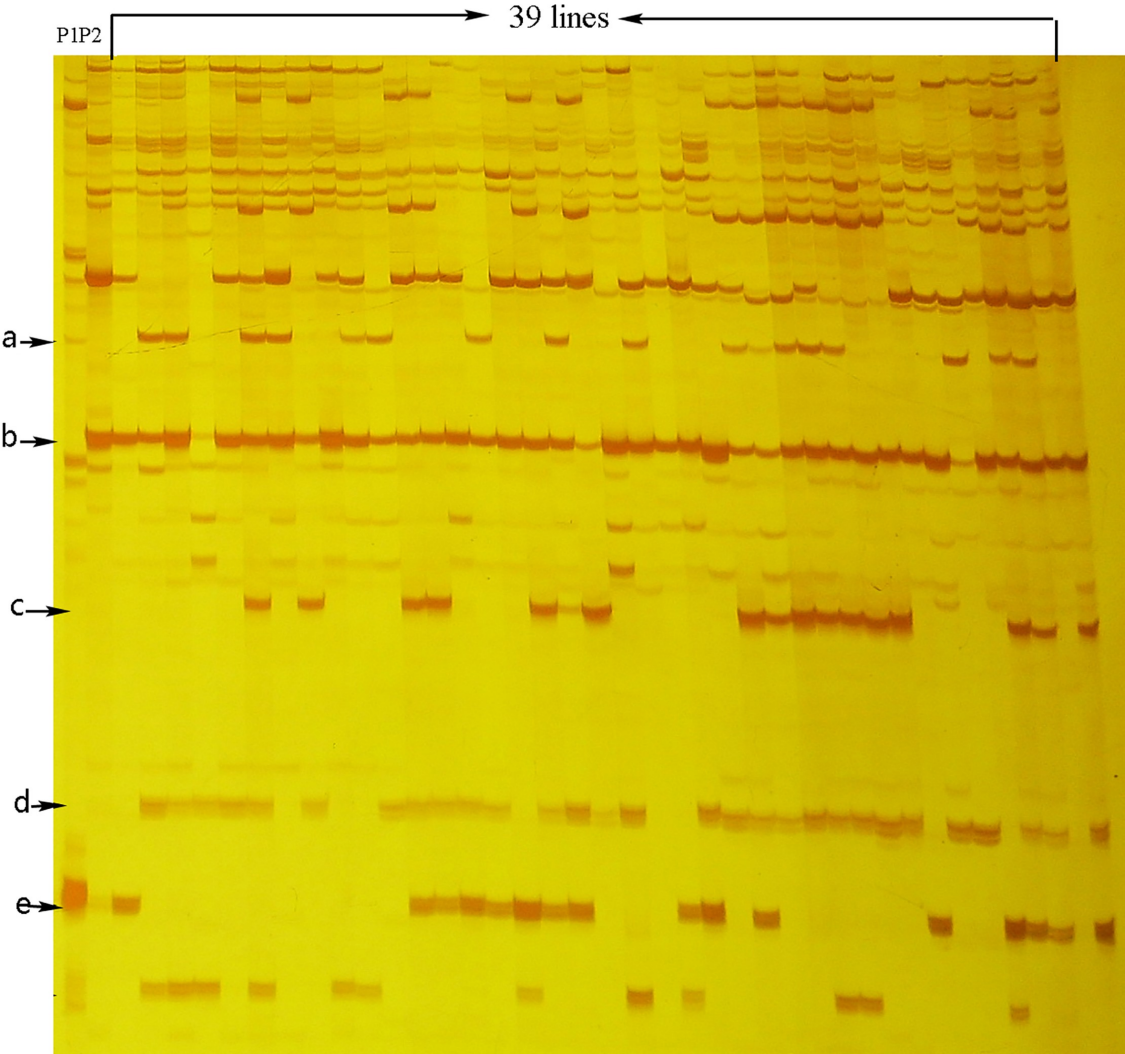
SRAP profiles of 39 lines as well as *B*.*rapa* and *O*. *violaceus* generated from one of the primer pairs (e1m6). Arrows of a, e indicated the specific bands for *O*. *violaceus*, b indicated the specific band for *B*. *rapa*, c,d indicated that the new bands not detected in the two parents.

To investigate the genomic composition of different lines and the introgression of alien functional genes from *O*. *violaceus*, we employed SRAP markers, as they are designed to amplify open reading frames (ORFs) [36]. Using 21 pairs of SRAP primers, 223 and 279 bands were amplified in *B*. *rapa* and *O*. *violaceus*, respectively. In addition, 191 bands were specific for *B*. *rapa*, 135 bands were specific for *O*. *violaceus*, and 88 bands were shared by both species. These lines produced 223–367 bands, with an average of 321, including specific bands for the two parents, bands shared by both parents, and new bands not detected in the two parents (Fig 6). On average, these lines contained 40.7% (36.6–45.7%) *B*. *rapa*-specific bands, 8.8% (6.5–11%) *O*. *violaceus-*specific bands, 23.3% (19.8–26.1%) shared bands, and 27.1% (24.1–29.6) new bands (S3 Table). Four *B*. *rapa*-specific bands were lost in some lines, three *B*. *rapa-*specific bands were present in all lines, and seven *O*. *violaceus-*specific bands were detected. We excised all these types’ bands and re-amplified by corresponding primers for sequencing. In general, only one PCR product was found in the bands excised from *B*. *rapa* and its progeny lines, whereas more than one product was usually found in the bands from *O*. *violaceus*. The sequences of the same band excised from *B*. *rapa* and various progeny lines were usually the same. The sequences of all *B*. *rapa-*specific bands were mapped successfully to the referenc genome of *B*. *rapa* (Chiifu-401-42, V 2.0), but the sequences of the new bands shared higher similarity with sequences from the C genome (*B*. *oleracea* var. capitata line 02–12, V1.1). However, only four bands contained sequences homologous to known functional genes (S4 Table). To investigate the seven *O*. *violaceus*-specific bands, 20 lines were subjected to band excision, but only one line had the same sequences as those of *O*. *violaceus*, which were homologous to the glutamate-ammonia ligase gene from *Arabidopsis* (S4 Table).

There was no obvious association between the genomic compositions and phenotypes of different lines or between their genomic compositions and chromosomal complements, as lines with different chromosomal complements had very similar band components (Table 1; S3 Table). Finally, the number of *O*. *violaceus*-specific bands was not obviously related to the degree of expression of the phenotypic traits of this species.

## Discussion

Although the lines examined in this study were selected from the progeny of a single intergeneric hybrid between *B*. *rapa* and *O*. *violaceus*, our molecular cytogenetics and genomic sequence analyses clearly demonstrated that they contained C-genome chromosomes and maintained *B*. *napus*-like chromosomal complements. This finding suggests that the hybrid or progeny was accidentally pollinated by pollen from *B*. *napus* plants that grew nearby, giving rise to progeny with the AAC genomes plus some *O*. *violaceus* chromosomes (Fig 1). Therefore, these lines were derived from the progenitor progeny by chromosomal reorganization, stabilization, and introgression of the alien fragments from *O*. *violaceus* during the relatively long process of artificial selection. The recovery of *B*. *napus*-like chromosomal complements from the progeny of the progenitor plants was not unexpected, as new *B*. *napus* lines are frequently selected from the progeny of interspecific hybrids between *B*. *napus* and *B*. *rapa* [38]. The predominant maintenance of the *B*. *napus*-like chromosomal complements in these lines also indicates that such plants had improved survival rates under selection for viability and fertility.

*Brassica* aneuploids have previously been identified from among the progeny of interspecific hybrids, such as one trisomic *B*. *rapa* line [22], one monosomic *B*. *napus* line [21], and one nullisomic *B*. *juncea* line [23]. However, using new cytogenetic tools to identify all homoeologous chromosomes, a high degree of aneuploidy together with inter- and intragenomic rearrangements was revealed in the successive generations of resynthesized *B*. *napus* [39]. Changes in the copy numbers of individual chromosomes appeared in early and later generations, while the mean chromosome number among lines was approximately 38. The reciprocal monosomy–trisomy of homoeologous chromosomes (1:3 copies) or nullisomy–tetrasomy (0:4 copies) primarily occurred within chromosome sets with extensive homoeology, i.e., A1/C1 and A2/C2. Therefore, dosage balance requirements maintained chromosome numbers at or near the tetraploid level. A similar pattern of aneuploidy by reciprocal loss and gain of homoeologous chromosomes was also found to prevail in the neo-allopolyploid species, *Tragopogon miscellus*, with a history of ca. 40 generations [40]. The protracted and prolonged chromosomal instability in the neo-allopolyploids and our hybrid progeny of advanced generations may increase the opportunity for changes to genome structure and gene expression [40]. In a previous study, seed yield and pollen viability were found to be inversely correlated with increasing aneuploidy, and lines that were additive for parental chromosomes showed the greatest viability [39]. The normal growth and high viability of our nulli-tetrasomic lines with 22 A-genome and 16 C-genome chromosomes also pointed to dosage balance and compensation between homoeologous A/C chromosomes, but the identity of the related chromosome set remains to be confirmed. Selection for growth vigor and fertility resulted in the preferential maintenance of lines with the complete complement of *B*. *napus* chromosomes, suggesting that plants with additive karyotypes were the best adapted of the lines.

The high frequency of homoeologous pairing of chromosomes in artificially synthesized *Brassica* allopolyploids together with non-paired and multipaired chromosomes [41–43] might lead to aberrant chromosome segregation and the formation of aneuploid gametes and, subsequently, aneuploid progeny, as the three cultivated *Brassica* diploids had closely related genomes. In our nulli-tetrasomic lines, in addition to the quadrivalents formed by the four A-genome chromosomes, other quadrivalents resulted from homeologous pairing between A- and C-genome chromosomes, showing non-diploidized cytological behavior. By contrast, quadrivalents from the four duplicated or different paralogs of A-genome chromosomes only occurred in ~15% of cells, indicating that they mainly formed two bivalents by homologous pairing. It was somewhat unexpected that most quadrivalents (~85%) consisted of chromosomes from both the A and C genomes. The variable frequencies of quadrivalent formation among lines points to subtle differences in chromosome homoeology and structure. The high frequency of quadrivalent formation in lines with 20 A-genome and 18 C-genome chromosomes also reveals the high degree of homoeology among some chromosome sets and indicates that the new *B*. *napus* lines required longer periods of time for meiotic diploidization.

Hybridization and introgression are important methods for the transfer and/or *de novo* origination of traits, and they play an important role in facilitating speciation [44] and plant breeding. Rapid introgression of alien genes/traits has been achieved using crosses that produce partial hybrids with the same chromosome number as the female parent but morphological variations in plants such as rice (*Oryza sativa*, [45]), sunflower (*Helianthus annuus*; [46]), and rapeseed [32,47–49]. Extensive stochastic genomic and epigenomic variations could be induced by the introgression of alien genetic elements [50]. Although few genes or DNA sequences from *O*. *violaceus* were confirmed to be incorporated in our lines, many more novel DNA bands were detected (S3 Table), which may provide the genetic foundation for their phenotypic variation (S1 Table). These *B*. *napus* introgression and aneuploid lines, particularly nulli-tetrasomics, are unique and should be valuable for studying the genetic control of meiotic pairing, chromosome balance, and dosage compensation, as well as chromosomal manipulation during breeding.

## Supporting information

S1 Fig Chromosome pairing in meiosis of hybrids between two lines and *B. rapa* /*B. napus* respectively. (DOCX)

S1 Table Leaf morphology and flowering time variation among lines. (DOCX)

S2 Table Chromosome pairing in meiosis of hybrids between four lines and *B. rapa /B. napus*. (DOCX)

S3 Table Genome compositions of various lines, as revealed by SRAP analysis. (DOCX)

S4 Table Sequence information for four types SRAP bands. (DOCX)
