# Supplementary material for: Phenotypic, cytogenetic, and molecular marker analysis of Brassica napus introgressants derived from an intergeneric hybridization with Orychophragmus
Source: PLoS One. 2019 Jan 10;14(1):e0210518. doi: 10.1371/journal.pone.0210518 (PMC6328085; doi:10.1371/journal.pone.0210518)
Supplement: S1 Fig — (DOCX) [file pone.0210518.s001.docx]

**
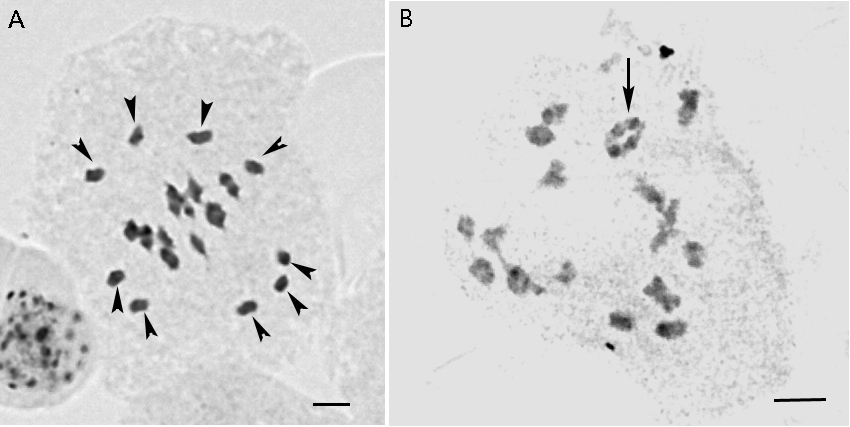
**

**S1 Figure**

**Chromosome pairing in meiosis of hybrids between two lines and *B. rapa*/**

***B. napus***

A. Chromosome paired as 10 bivalents and 9 univalents (arrowheads) in one pollen mother cell of the hybrid between line 6 and *B. rapa*, “Aijiaohuang”. B. One quadrivalent (Arrow) formed in one pollen mother cell of the hybrid between line 8 and *B. napus*, “Oro”. Bar=10µm.
