# Supplementary material for: Phenotypic, cytogenetic, and molecular marker analysis of Brassica napus introgressants derived from an intergeneric hybridization with Orychophragmus
Source: PLoS One. 2019 Jan 10;14(1):e0210518. doi: 10.1371/journal.pone.0210518 (PMC6328085; doi:10.1371/journal.pone.0210518)
Supplement: S1 Table — (DOCX) [file pone.0210518.s002.docx]

**Table S1**

**Leaf morphology and flowering time variation among lines**

| **Lines** | **Leaf Morphology** | | | | | | | **Flowering time** |
| --- | --- | --- | --- | --- | --- | --- | --- | --- |
|  | **Lobed leaves** | **Serrated leaves** | **Flat and**  **curly** | **Leaf**  **hairs** | **Leaf color** | **Petiole length (cm)** | **Petiole**  **angle (°)** | **Days** |
| **1** | N | Y | F | N | G | 39.3±0.6 | 43.3±2.9 | 135 |
| **3** | N | Y | F | N | G | 27.0±5.3 | 55.0±22.9 | 133 |
| **7** | N | N | W | N | G | 39.3±0.6 | 41.7±12.6 | 91 |
| **8** | N | N | F | N | LP | 40.8±1.6 | 86.7±2.9 | 172 |
| **9** | Y | Y | CW | N | LP | 32.7±5.5 | 40.0±10.0 | 185 |
| **10** | N | N | W | N | G | 27.3±6.5 | 31.7±2.9 | 188 |
| **11** | Y | Y | CW | Y | G | / | / | 162 |
| **16** | N | Y | F | N | G | 42.0±6.6 | 71.7±12.6 | 86 |
| **17** | N | Y | F | N | G | 38.7±2.5 | 45.0±5.0 | 164 |
| **20** | N | Y | CW | N | LP | 28.7±3.1 | 66.7±5.8 | 167 |
| **21** | Y | N | CW | N | P | 27.7±2.5 | 50.0±5.0 | 56 |
| **22** | Y | Y | CW | Y | G | 29.3±8.1 | 56.7±5.8 | 154 |
| **24** | N | Y | F | Y | G | 51.3±10.3 | 75.0±21.8 | 112 |
| **28** | N | Y | CW | N | G | 42.0±4.4 | 51.7±10.4 | 125 |
| **32** | N | Y | F | N | G | 31.7±4.7 | 57.3±17.8 | 124 |
| **33** | Y | Y | F | N | G | 43.0±2.6 | 68.3±10.4 | 74 |
| **35** | N | Y | F | Y | G | / | / | 163 |
| **37** | Y | Y | CW | N | LP | / | / | 165 |
| **38** | N | Y | CW | N | G | 37.7 ±2.5 | 55. 0±5.0 | 91 |
| **6** | Y | Y | F | N | LP | 40.7±1.5 | 86.7±5.8 | 89 |
| **13** | N | Y | CW | N | G | 34.7±4.0 | 58.3±7.6 | 62 |
| **14** | N | Y | CW | N | G | 37.3±2.1 | 80.0±17.3 | 66 |
| **15** | N | Y | CW | N | LP | 32.3±5.1 | 50.0±8.7 | 155 |
| **18** | N | Y | F | N | LP | 44.3±4.0 | 55.0±5.0 | 102 |
| **19** | Y | Y | F | N | G | 30.5±2.3 | 45.0±15.0 | 134 |
| **23** | N | Y | CW | N | LP | 34.8±6.7 | 58.3±12.6 | 150 |
| **25** | Y | Y | CW | N | G | 45.2±12.0 | 51.7±10.4 | 46 |
| **26** | N | Y | CW | N | G | 31.0±3.6 | 43.3±7.6 | 153 |
| **27** | Y | Y | F | N | LP | 39.7±5.7 | 45.7±12.5 | 175 |
| **29** | N | Y | F | N | G | 35.0±3.0 | 51.7±10.4 | 143 |
| **30** | N | Y | F | N | G | 28.7±3.2 | 63.3±20.8 | 164 |
| **31** | Y | N | CW | N | G | 43.3±3.8 | 81.7±10.4 | 166 |
| **34** | Y | Y | F | N | G | 27.3±9.3 | 58.3±2.9 | 112 |
| **39** | Y | Y | F | N | G | 38.7 ±5.7 | 70.0± 10.0 | 60 |
| **2** | N | N | CW | N | P | 44.0±3.6 | 86.7±5.8 | 176 |
| **5** | Y | Y | F | N | G | 37.5±3.0 | 88.3±2.9 | 137 |
| **12** | Y | Y | CW | N | G | 39.8±3.0 | 45.0±18.0 | 157 |
| **4** | N | Y | F | Y | G | 39.0±10.6 | 55.0±0 | 132 |
| **36** | Y | Y | F | Y | LP | 48.7±8.1 | 46. 7±15.3 | 117 |

Note: Y=Yes；N=No；F=Flat；CW=Curly and Wizened；LP=Light Purple；P=Purple
