# Supplementary material for: Phenotypic, cytogenetic, and molecular marker analysis of Brassica napus introgressants derived from an intergeneric hybridization with Orychophragmus
Source: PLoS One. 2019 Jan 10;14(1):e0210518. doi: 10.1371/journal.pone.0210518 (PMC6328085; doi:10.1371/journal.pone.0210518)
Supplement: S2 Table — (DOCX) [file pone.0210518.s003.docx]

**S2 Table**

**Chromosome pairing in meiosis of hybrids between four lines and *B. rapa* /**

***B. napus***

| Lines | Chromosome composition  (A/C) | Chromosome pairing in meiosis of hybrids with *B. rapa* | | | | | Chromosome pairing in meiosis of hybrids  with *B. napus* | | |
| --- | --- | --- | --- | --- | --- | --- | --- | --- | --- |
|  |  | 9II+  11I | 10II+9I | 11II+7I | 12II+5I | Total cells | 19II | 17II+1IV | Total cells |
| 6 | 20/18 | 1 | 25 | 4 | 1 | 31 | 20 | 0 | 20 |
| 13 | 20/18 | 0 | 10 | 3 | 0 | 13 | 28 | 2 | 30 |
| 7 | 22/16 | 1 | 21 | 11 | 2 | 35 | 57 | 27 | 84 |
| 8 | 22/16 | 5 | 23 | 7 | 1 | 36 | 19 | 6 | 25 |

Note: I represented univalents; II represent bivalents.
