# Supplementary material for: Phenotypic, cytogenetic, and molecular marker analysis of Brassica napus introgressants derived from an intergeneric hybridization with Orychophragmus
Source: PLoS One. 2019 Jan 10;14(1):e0210518. doi: 10.1371/journal.pone.0210518 (PMC6328085; doi:10.1371/journal.pone.0210518)
Supplement: S3 Table — (DOCX) [file pone.0210518.s004.docx]

**S3 Table. Genome compositions of various lines revealed by SRAP analysis**

| Lines | *O. violaceus* specific bands |  | *B. rapa* Specific bands |  | Shared bands |  | New bands |  | Total bands |
| --- | --- | --- | --- | --- | --- | --- | --- | --- | --- |
|  | Number | Percent  (%) | Number | Percent  (%) | Number | Percent  (%) | Number | Percent  (%) | Number |
| P1 | 135 |  |  |  | 88 |  |  |  | 223 |
| P2 |  |  | 191 |  | 88 |  |  |  | 279 |
| 1 | 30 | 9.4 | 122 | 38.4 | 83 | 26.1 | 83 | 26.1 | 318 |
| 3 | 30 | 9.7 | 122 | 39.6 | 73 | 23.7 | 83 | 26.9 | 308 |
| 7 | 32 | 9.8 | 140 | 42.7 | 78 | 23.8 | 78 | 23.8 | 328 |
| 8 | 30 | 9 | 131 | 39.2 | 74 | 22.2 | 99 | 29.6 | 334 |
| 9 | 28 | 8.9 | 143 | 45.7 | 62 | 19.8 | 80 | 25.6 | 313 |
| 10 | 31 | 9.4 | 134 | 40.5 | 70 | 21.1 | 96 | 29 | 331 |
| 11 | 38 | 11 | 132 | 38.3 | 77 | 22.3 | 98 | 28.4 | 345 |
| 16 | 26 | 8.2 | 136 | 43 | 76 | 24.1 | 78 | 24.7 | 316 |
| 17 | 27 | 8 | 137 | 40.7 | 79 | 23.4 | 94 | 27.9 | 337 |
| 20 | 37 | 10.7 | 140 | 40.5 | 77 | 22.3 | 92 | 26.6 | 346 |
| 21 | 21 | 6.8 | 127 | 41.2 | 75 | 24.4 | 85 | 27.6 | 308 |
| 22 | 30 | 10.1 | 117 | 39.4 | 75 | 25.3 | 75 | 25.3 | 297 |
| 24 | 27 | 8.5 | 127 | 39.8 | 77 | 24.1 | 88 | 27.6 | 319 |
| 28 | 35 | 9.3 | 150 | 39.7 | 81 | 21.4 | 112 | 29.6 | 378 |
| 32 | 21 | 6.8 | 133 | 43 | 72 | 23.3 | 83 | 26.9 | 309 |
| 33 | 32 | 9.9 | 127 | 39.3 | 77 | 23.8 | 87 | 26.9 | 323 |
| 35 | 30 | 9.2 | 134 | 41 | 80 | 24.5 | 83 | 25.4 | 327 |
| 37 | 33 | 9.1 | 153 | 42.3 | 80 | 22.1 | 96 | 26.5 | 362 |
| 38 | 22 | 7.2 | 131 | 43 | 79 | 25.9 | 73 | 23.9 | 305 |
| Average | 29.5 | 9.0 | 133.5 | 40.9 | 76.1 | 23.3 | 87.5 | 26.8 | 326.5 |
| 6 | 31 | 9.4 | 136 | 41.2 | 75 | 22.7 | 88 | 26.7 | 330 |
| 13 | 25 | 7.6 | 132 | 40.4 | 77 | 23.5 | 93 | 28.4 | 327 |
| 14 | 32 | 9.5 | 135 | 39.9 | 79 | 23.4 | 92 | 27.2 | 338 |
| 15 | 35 | 10.5 | 127 | 38.1 | 74 | 22.2 | 97 | 29.1 | 333 |
| 18 | 31 | 9.4 | 138 | 41.7 | 79 | 23.9 | 83 | 25.1 | 331 |
| 19 | 28 | 8.9 | 131 | 41.7 | 74 | 23.6 | 81 | 25.8 | 314 |
| 23 | 30 | 8.9 | 140 | 41.4 | 78 | 23.1 | 90 | 26.6 | 338 |
| 25 | 26 | 7.9 | 129 | 39.2 | 80 | 24.3 | 94 | 28.6 | 329 |
| 26 | 16 | 6.5 | 112 | 45.5 | 59 | 24 | 59 | 24 | 246 |
| 27 | 24 | 7.8 | 125 | 40.7 | 71 | 23.1 | 87 | 28.3 | 307 |
| 29 | 36 | 10.1 | 137 | 38.4 | 88 | 24.6 | 96 | 26.9 | 357 |
| 30 | 29 | 8.9 | 124 | 37.9 | 79 | 24.2 | 95 | 29.1 | 327 |
| 31 | 27 | 9.1 | 125 | 42.1 | 53 | 17.8 | 92 | 31 | 297 |
| 34 | 25 | 7.9 | 127 | 39.9 | 74 | 23.3 | 92 | 28.9 | 318 |
| 39 | 24 | 6.9 | 141 | 40.4 | 81 | 23.2 | 103 | 29.5 | 349 |
|  |  |  |  |  |  |  |  |  |  |
| Average | 27.9 | 8.6 | 130.6 | 40.6 | 74.7 | 23.1 | 89.5 | 27.7 | 322.7 |
| 2 | 35 | 10.2 | 126 | 36.6 | 79 | 23 | 104 | 30.2 | 344 |
| 5 | 31 | 9.7 | 131 | 40.8 | 78 | 24.3 | 81 | 25.2 | 321 |
| 12 | 27 | 8 | 139 | 41.4 | 77 | 22.9 | 93 | 27.7 | 336 |
| 4 | 30 | 10 | 126 | 42.1 | 71 | 23.7 | 72 | 24.1 | 299 |
| 36 | 27 | 8.1 | 135 | 40.5 | 78 | 23.4 | 93 | 27.9 | 333 |
| Average | 30 | 9.2 | 131.4 | 40.28 | 76.6 | 23.46 | 88.6 | 27.02 | 326.6 |
