# Supplementary material for: Phenotypic, cytogenetic, and molecular marker analysis of Brassica napus introgressants derived from an intergeneric hybridization with Orychophragmus
Source: PLoS One. 2019 Jan 10;14(1):e0210518. doi: 10.1371/journal.pone.0210518 (PMC6328085; doi:10.1371/journal.pone.0210518)
Supplement: S4 Table — (DOCX) [file pone.0210518.s005.docx]

**S4 Table. Sequence information for four type’s SRAP bands**

|  | **Total bands** | **Fragment length** | **Location in *B. rapa* genome or *B. oleracea* genome/Identities** | **Gene identification** |
| --- | --- | --- | --- | --- |
| ***B. rapa* bands lost in some of the lines using three primer pairs** | | | | |
| **L1*** | 2 | 328 | A genome Scaffold000141  Identities =326/328 (99%) | AT1G65930.1; isocitrate dehydrogenase |
| **L1*** | 2 | 328 | A genome Scaffold000157  Identities =326/327 (99%) | No |
| **L2** | 3 | 237 | A10 Identities= 237/237(100%) | gi\|150936845\|gb\|ES967219.1\|  *Brassica napus* cDNA |
| **L3** | 4 | 223 | A genome Scaffold000104; Identities = 221/223 (99%) | No |
| **L4** | 3 | 102 | A03 Identities = 102/102 (100%) | AT4G31300.1; endopeptidase |
| **New bands for two parents found in some of lines using six primer pairs** | | | | |
| **N1** | 4 | 181 | C06#2011-08-02#BGI  Identities = 102/106 (96%) | No |
| **N2** | 3 | 95 | C02#2011-08-02#BGI  Identities = 95/95 (100%) | No |
| **N3** | 3 | 281 | C07#2011-08-02#BGI  Identities = 134/135 (99%) | AT1G65650.1, ubiquitin thiolesterase |
| **N4** | 3 | 270 | C07#2011-08-02#BGI  Identities = 144/145 (99%) | AT1G65650.1; ubiquitin thiolesterase |
| **N5** | 3 | 154 | C01#2011-08-02#BGI  Identities = 83/83 (100%) | No |
| **N6** | 3 | 133 | C02#2011-08-02#BGI  Identities = 126/133 (94%) | No |
| **N7** | 4 | 274 | Scaffold000444  Identities = 224/226 (99%) | No |
| ***B. rapa* specific bands found in all lines from using three primer pairs** | | | | |
|  | | | | |
| **BS1** | 4 | 236 | >A01  Identities = 233/236 (98%) | No |
| **BS2** | 3 | 322 | >A09 Identities = 313/322 (97%) | No |
| **BS3** | 4 | 314 | >A09  Identities = 303/314 (96%) | No |
| ***O. violaceus* specific bands found in some of lines using 7 primer pairs** | | | | |
| **OS1** | 4 | 319 |  | AT3G17820.1 glutamate-ammonia ligase |
| **OS2-7** | 16 |  | No same sequence found in progenies as in *O. violaceus* |  |

* Two types of sequences were present in this band.
